# These are all our lanes

**DOI:** 10.1186/s40621-019-0199-6

**Published:** 2019-05-29

**Authors:** Lois K. Lee

**Affiliations:** Boston Children’s Hospital, Division of Emergency Medicine, Boston, MA 02115 USA

Last fall there was a robust physician social media response about the medical community’s role in firearm injury prevention with #ThisIsOurLane. And now there is greater attention being paid to preventing firearm injuries, whether intentional or unintentional. In our work as medical providers, we have experienced first hand the challenges of caring for patients with gunshot wounds and the anguish of comforting their families. As clinicians, injury prevention specialists, and public health advocates we understand the importance of a multi-pronged approach to prevent firearm injuries. This has been and will continue to be our lane.

As an injury prevention professional organization, the Injury Free Coalition for Kids® strives to decrease injuries to children and adolescents from all mechanisms of injury, unintentional and intentional. We work as clinicians caring for those who have sustained injuries and as public health advocates trying to decrease injuries. At the 2019 Injury Free Coalition for Kids® Annual Meeting, we covered a broad range of injury prevention topics, ranging from motor vehicle crashes to dog bites to firearms. We described innovative programs and discussed best practices. Reflecting the range of topics presented at the conference, this annual supplement for the 2019 Injury Free Coalition for Kids® meeting include articles on a variety of injury prevention areas.

This year at the conference we had a morning session focused on safe sleep, to highlight infant sleep-related death, a persistently leading cause of infant mortality in the U.S. Preventing Sudden Unexpected Infant Deaths (SUIDs) in infants with safe sleep practices is our lane. In this supplement we have included two articles focused on Safe Sleep. One focuses on associated risk factors for unintentional sleep-related infant deaths, including geographic hot spots, in Cook County, Illinois. A second article describes a quality improvement program to improve safe sleep practices on a hospital inpatient unit. In addition to safe sleep, this supplement includes motor vehicle safety related research highlighting gaps in child rear-facing car seat use in crashes and marijuana use in teen drivers. Preventing motor vehicle crash deaths and injuries is our lane. Another article describes a quality improvement program for injury prevention anticipatory guidance and screening for social determinants of health in primary care pediatric practices. Providing anticipatory guidance to prevent injuries is our lane. In addition, we include an article about pediatric traumatic brain injuries, focusing on changes in the mechanism of injury over a 10-year period. Preventing traumatic brain injuries is our lane.

Finally, this supplement also includes two articles related to firearm injuries. One study examines firearm injury prevention education as part of a medical school curriculum. Another study analyzed social workers’ determination of neglect in scenarios where children have access to a firearm, stratifying by type of Child Access Prevention (CAP) laws. Preventing firearm injuries is our lane.

Injuries continue to be the leading cause of death and disability to children and adolescents in developed countries. But many of these injuries are preventable with safer equipment, effective legislation, and public awareness. In the world of injury prevention, there is much work that must still be done. That is why these are all our lanes.

